# Book Reviews

**Published:** 1914-12

**Authors:** 


### BOOK REVIEWS

Books mentioned may be inspected at and ordered through this office. So far as possible, books received in any month will be reviewed in the issue of the second month following.

**We venture to suggest to publishers that it is difficult to arrange shelf room economically or aesthetically when books are of such different sizes. It occurs to us that, so far as medical books are concerned, provision could be made for all requirements, from cyclopaedias down to pocket manuals in five standard sizes, each of the same height and depth.**

**Municipal Ordinances, Rules and Regulations Pertaining to Public Health.** Adopted during 1912 by cities of the U. S. having a population of over 10,000 in 1910. Reprint No. 199 from the Public Health Reports, U. S. Public Health Service.

This is valuable for reference but we are pleased to note that few new ordinances have been enacted by any of the cities in our territory.

**The Practitioner's Visiting List for 1915.** Four styles: weekly, monthly, perpetual, sixty-patient. Pocket size; substantially bound in leather with flap, pocket, etc.; $1.25, net. Lea & Febiger, Publishers, Philadelphia and New York.

This visiting list is standard and requires no detailed description. We are pleased to receive a copy in time for review before the new year begins.

**A Manual of Biologic Therapeutics,** Sera, Bacterins, Phylacogens, Tuberculins, Glandular Extracts, Toxins, Cultures, Antigens, etc. Parke, Davis & Co. 174 pages, illustrated.

The importance of this field of therapeutics is now fully realized. One of the most important problems connected with it, is the practical availability of means to carry out theoretic indications. To prevent misconceptions, it should be stated that this work is far more than a price list or trade circular. In fact, it is not a price list at all. It is a frank, scientific, comparatively brief statement of what the biologic preparations are, how they are made, how they are used, what they accomplish and the limitations and contraindications to the general theories of their action. (See more detailed description in Publicity Dept.)

**Manual of Obstetrics.** By Edward P. Davis, A. M., M. D., Professor of Obstetrics in the Jefferson Medical College, Philadelphia. 12mo of 463 pages, 171 illustrations. Philadelphia and London: W. B. Saunders Company, 1914. Cloth, $2.25 net.

This work is a happy mean between the Quiz Compend or other condensed students' manual and the exhaustive treatise. It is authoritative, thoroughly modern, portable, so that it may be read to while away the hours of waiting at obstetric cases, and well adapted to the needs of the practitioner.

---

**An Epitome of Pediatrics.** By Henry Enos Tuley, A. B., M. D., Late Professor of Obstetrics, Medical Department, University of Louisville; Editor Louisville Monthly Journal of Medicine and Surgery; Late Chairman of Section Diseases of Children, American Medical Association; Ex-President American Association Medical Milk Commissions, etc. New (2d) edition, revised and enlarged. 12mo, 324 pages. Cloth, $1.00, net. Lea & Febiger, Publishers, Philadelphia and New York, 1914. (Lea's Series of Medical Epitomes.)

The appending of questions at the end of each chapter and of selected State Board questions at the end of the book, gives this series whatever value attaches to quiz compends, without interfering with the logical presentation of the subject matter in ordinary didactic form. Paediatrics is a branch of medicine in which notable advances have been made and the author has included these in his book.

---

**Report of the Dept. of Pathology and Dept. of Clinical Psychiatry** of the Central Indiana Hospital for the Insane? 1911-12 and 1912-13. (Vol. 5.)

This contains, beside the usual statistics, various papers and clinical reports of general interest.

---

**A Text-Book of the Diseases of the Nose and Throat.** By Jonathan Wright, M. D., Director of the Department of the Laboratories, New York, Post-Graduate Medical School and Hospital, and Harmon Smith, M. D., Surgeon to Throat Department of the Manhattan Eye, Ear, Nose and Throat Hospital; Clinical Professor of Laryngology and Rhinology, Cornell University Medical School. Octavo, 683 pages, with 313 engravings and 14 plates. Cloth, $5.00, net. Lea & Febiger, Publishers, Philadelphia and New York, 1914.

The first chapter, of considerable length and well illustrated describe the standard equipment of the office. The work is systematic and complete, even going to gastroscopy, bronchoscopy, oesophageal diseases and exanthematous lesions of the mucous membranes. What impresses us particularly is the lucid discussion of physiology for each organ and the inclusion of a great deal of interesting and useful information of general relations to diseased conditions, which might have been omitted without being missed and yet which is introduced in such a way as to appear natural to the subject. It is this factor in authorship which makes a book worth while, by stimulating new ideas.

---

**Practical Bandaging**, Eldridge L. Eliason, A. B., M. D., Philadelphia, published by the J. B. Lippincott Co. 124 pages, 147 illustrations, $1.50.

This is a neat and complete treatise on the subject.

---

**Life and Law**, The Development and Exercise of the Sex Function, together with a Study of the Effects of Certain Natural and Human Laws and a Consideration of the Hygiene of Sex, by Maude Glasgow, M. D., New York, published by G. P. Putnam's Sons, 194 pages, $1.25.

This is a good presentation of the subject for the laywoman though, like most such books, even by male authors, it gives the impression of a feministic point of view. We fully agree with the statement that "all the statutes which safe-guard one sex at the expense of another breed and foster immorality" but we certainly do not agree with the implication that the male sex is safe-guarded at the expense of the female. So far as we are aware, there is no state in the country which does not make a sincere effort to avert crimes against sex and, except with regard to regulations of prostitutes and especially street walkers, we cannot think of any law in which the male sex is protected in this sense. That the laws do not always work out satisfactorily is inevitable in any case. For instance, we recall a case in which a grand jury reluctantly discharged a man because the girl obstinately stuck to statements, regarding dates, etc., which were absurdly impossible. Jane Addams is quoted as saying that the "punishment for rape is the same as that inflicted for $15 worth of property." Such a statement is palpably untrue except possibly in the sense that as the law gives judges great latitude in the matter of penalties, to allow for extenuating and intensifying circumstances and as this privilege is sometimes abused, as in regard to penalties for violation of traffic ordinances, one might find instances in which a particularly dastardly theft of $15 has been more severely punished than the rape of a woman who had led on a youth up to a certain point, and who had not a clear record herself. Except in such instances as have been mentioned, we fully agree with the author but the exceptions are important both as indicating a biased view as to relative standards of male and of female morality and a failure to grasp the fundamental principle that law should not extend privileges to a sex or to any other class but should depend upon absolute facts of right and wrong.

---

**Handbook of Pharmacology,** By Charles W. Greene, A. B., A. M., Ph. D. Octavo, 420 pages, illustrated by 70 engravings in black and colors. Extra muslin, $3.50, net. Wm. Wood & Co., Publishers, New York.

A work of this sort tends to place medical practice upon a firm scientific basis and to eliminate the survivals of empiricism more or less justly charged against the strictly medical practitioner. We often speak scornfully of ''drugging a patient.'' As a matter of fact, it is just as logical to treat a chemic disease by chemic methods as a mechanic lesion by mechanic methods. And, even if a pathologic condition is not chemic in the direct sense, if it can be removed or ameliorated by a demonstrated action of a drug, the therapeusis is just as logical and scientific as if any other means were employed. Pharmacology is one of the most important branches of medicine and, on account of the rapidly increasing amount of definite experimental knowledge now available, it now deserves specialized consideration, apart from the details of materia medica and even from its ultimate application in what is known as therapeutics in the limited sense. While the time is not ripe for a work of such completeness as may be written in some lines of medical science, Greene has presented a thorough and logical treatise, of surprisingly large scope and has dealt with the subject in such a way as to facilitate the extension of study to future developments. We bespeak for the book, especially the attention of those practitioners who realize that their knowledge of just how drugs act, is somewhat vague. Those who have already devoted attention to this subject will realize the desirability of extending their knowledge so far as possible.

---

**Practical Homoeopathic Therapeutics,** Dr. W. A. Dewey, Prof. of Materia Medica in the Homoeopathic Medical College of the University of Michigan, Ann Arbor: 2d edition, 426 pages, Boericke & Tafel, Philadelphia.

Very wisely, the author omits pathology, diagnosis, etc., and confines himself to a statement in concise terms of the therapeutic indications, from the homoeopathic standpoint. The simplicity and definiteness of the system should appeal strongly to the homoeopathic practitioner while it arouses the envy of those not thus affiliated.

---

**The Pharmacy Handbook,** F. W. Crossley-Holland, F. C. S., London, Associate Editor of the Prescriber; 224 pages, $2.00, Oxford University Press, 35 W. 32, N. Y. (American Branch)

The purpose of this work is to supplement the technical knowledge of the pharmacist in materia medica, by the discussion of therapeutic uses, a description of the newer materia medica, especially in serums, phylacogens, hormones and other biologic products, etc. Diet, balecology, anaesthetics, hydrotherapy, pharmaceutic bacteriology are discussed and there is an excellent chapter on ethics and a book list.

---

**Motherhood,** pamphlet, 30 pages, 10 cents per copy, purchaser's name and address on front page if ordered in lots of 25 or more. Author and publisher, Dr. E. S. Harris, Independence, Mo.

This pamphlet contains advice to the mother regarding the care of the baby and a brief consideration of her own case.

---

**Nervous and Mental Diseases.** By Joseph Darvin Nagel, M. D., Consulting Physician to the French Hospital of New York, Member of New York Academy of Medicine, Honorary Member Societe Royal de Belique, etc., Physician to St. Chrysostom's Dispensary. New (2d) edition, revised and enlarged, 12 mo., 293 pages, with 50 engravings and a colored plate. Cloth, $1.00, net. (The Medical Epitome Series.) Lea & Febiger, Publishers, Philadelphia and New York, 1914.

One of the most important items in this book is the colored plate showing positive and negative Wasserman tests. While we do not feel competent to pass judgement on the accuracy of this test, the literature plainly shows that much confusion of diagnosis has occurred from wrong conclusions from doubtful reactions. Whether typic plus and minus reactions are absolutely reliable is sub judice but, at any rate, the percentage of error may be very much reduced if the physician in charge of a case has clearly in mind, just how these reactions appear in the test tube. The work is divided anatomically—peripheral nervous system, cord, medulla, cerebrum and meninges and then, contrary to logical theory but very necessary practically, Psychiatry (Diseases of the Mind) is separately discussed. Practical efficiency is further served by discussing insanity under such headings as "imperfect development of the brain," abnormal brain organization, anaemia and hyperaemia, "insanity due to microscopic structural alterations," toxic substances, developmental changes and, finally, rarer forms of insanity are discussed. Questions follow each chapter, to insure the mental grasp of the student.

---

**Medical Dictionary for Nurses**, Amy Elizabeth Pope, San Francisco, 288 pages, $1.00, G. P. Putnam's Sons, New York.

This is a conveniently abridged dictionary, with pronunciation and derivation and very clear definitions. Tables of abbreviations used in prescription writing, of the elements, poisons, composition of food stuffs, etc., are appended. Considering the size and price of the work, it is surprising that the abridgement is not more conspicuous.

---

**Pathogenic Microörganisms.** (Including Bacteria and Protozoa.) A Practical Manual for Students, Physicians and Health Officers. By William H. Park, M. D., Professor of Bacteriology and Hygiene in the University and Bellevue Hospital Medical College, and Director of the Bureau of Laboratories of the Department of Health, New York City, and Anna W. Williams, M. D., Assistant Director of the Bureau of Laboratories, New York City; Consulting Pathologist to the New York Infirmary for Women and Children. New (5th) edition, thoroughly revised. Octavo, 684 pages, with 210 illustrations and 9 full-page plates, Cloth, $4.00, net. Lea & Febiger, Publishers, Philadelphia and New York, 1914.

Part I, Principles of Microbiology, deals with classification, cultivation, obtaining material from diseased human beings, immunity, etc., 228 pages. Part II, Pathogenic Microörganisms Individually Considered, gives historic notes and discusses in detail, the characteristics, infectivity, therapeutic use of vaccines, etc., up to page 598. Part III, Applied Microbiology, assembles many of the principles previously discussed as applied to examinations of water, air, soil, sewage disposal, milk, disinfection, etc. One of the most interesting items of the work is the analytic chart prepared by the Committee on means of identification of Bacterial Species of the Society of American Bacteriologists. This gives a glossary of terms, the decimal system of indicating characteristics of morphology, growth, etc., (after the analogy of the decimal system of cataloguing books) and a check system for filling in a blank report. The decimal system impresses us so favorably that we regret that the authors have not applied it in practice in the second part. With the aid of excellent index, it would seem that almost every theoretic and practical point in regard to infections can be found in this work. Thus, it is available not only for the technical bacteriologist but for the sanitarian, the diagnostician, the worker with vaccines, serums, etc.,—not to omit its value to the general practitioner as a means of keeping in touch with what may be accomplished by the specialist.

---

**Medical Directory of N. Y., N. J. and Conn.** issued by the Medical Society of the State of N. Y., October 1, 1914.

This directory is familiar to all and is one of the most practical co-operative undertakings of the profession. We urge our readers to use it, not only as a reference book but to keep in touch with old acquaintances of whom they may have lost sight. A total of 18,210 physicians are listed, 14,114 for N. Y. State, 7,724 for Greater N. Y. City, the excess over 1913 being respectively, 352, 337 and 297. These increases are not numerically important and scarcely correspond to the increase of population but it is to be regretted that any excess at all is shown. The directory reflects great credit on the diligence and accuracy of the compilers. We bespeak the assistance of our readers in accumulating information as to changes of address, deaths, removals, etc.

---

**Selected Papers, Surgical and Scientific,** by Roswell Park. Compiled by Drs. Charles G. Stockton and Edgar R. McGuire and edited by Julian Park. 381 pages, $3.00, published by the Courier Co., of Buffalo.

In contrast to most memorial works, this book is given especial scientific and professional interest by the fact that it deals only briefly with biography and omits almost entirely the eulogistic matter that there is strong temptation to expand unduly. To the memory of no man more than to Roswell Park, is this conception of a memorial more appropriate. He was a man of too fine sentiment to be sentimental. His own works praise him more eloquently than can the words of the most eloquent writer and because they are, themselves, lacking in the personal, introspective element but show a brilliant mind concentrated on some practical problem and illuminated from every possible aspect by a clear scientific understanding.

Dr. Stockton has prefaced the work with a brief biography and family history which exemplifies the two cardinal excellencies of historic writing—a clear and accurate presentation of facts and the logical relation of those facts to one another and to environment and circumstances. We wish that every one who considers history a succession of accidents and a valuable and successful life as luck, or who has no sense of gratitude to his forebears, might read this memoir. It will give a broad meaning to the fifth commandment that, in this country, we are too prone to ignore.

From the large but scarcely prolific writings of Dr. Park, various papers have been selected, from one on Maternal Impressions in 1879 to the very last of his writings, Of What Does the Universe Consist? published in this Journal last March. His article on the Status of Antiseptic Surgery, in 1882, is noteworthy historically, as being one of the first in America and even more so for indicating a clear conception of general principles which have outlived details of practice then in vogue. Most of the papers are distinctly technical but several have been included to illustrate Dr. Park's grasp of general scientific theory and power of literary focussing. And, ultimately, we are not sure but that these latter selections are as practical as the former.

